# Adoption of the concept of person-centred care into discourse in Europe: a systematic literature review

**DOI:** 10.1108/JHOM-01-2021-0008

**Published:** 2021-09-13

**Authors:** Kristina Rosengren, Petra Brannefors, Eric Carlstrom

**Affiliations:** Institute of Health and Care Sciences, University of Gothenburg , Goteborg, Sweden; Health Management, Health Care Sciences , Gothenburg, Sweden

**Keywords:** Beveridge, Bismarck, Europe, Evidence-based, Literature review, Person-centred care

## Abstract

**Purpose:**

This study aims to describe how person-centred care, as a concept, has been adopted into discourse in 23 European countries in relation to their healthcare systems (Beveridge, Bismarck, out of pocket).

**Design/methodology/approach:**

A literature review inspired by the SPICE model, using both scientific studies (CINHAL, Medline, Scopus) and grey literature (Google), was conducted. A total of 1,194 documents from CINHAL ( *n* = 139), Medline ( *n* = 245), Scopus ( *n* = 493) and Google ( *n* = 317) were analysed for content and scope of person-centred care in each country. Countries were grouped based on healthcare systems.

**Findings:**

Results from descriptive statistics (percentage, range) revealed that person-centred care was most common in the United Kingdom ( *n* = 481, 40.3%), Sweden ( *n* = 231, 19.3%), the Netherlands ( *n* = 80, 6.7%), Northern Ireland ( *n* = 79, 6.6%) and Norway ( *n* = 61, 5.1%) compared with Poland (0.6%), Hungary (0.5%), Greece (0.4%), Latvia (0.4%) and Serbia (0%). Based on healthcare systems, seven out of ten countries with the Beveridge model used person-centred care backed by scientific literature ( *n* = 999), as opposed to the Bismarck model, which was mostly supported by grey literature ( *n* = 190).

**Practical implications:**

Adoption of the concept of person-centred care into discourse requires a systematic approach at the national (politicians), regional (guidelines) and local (specific healthcare settings) levels visualised by decision-making to establish a well-integrated phenomenon in Europe.

**Social implications:**

Evidence-based knowledge as well as national regulations regarding person-centred care are important tools to motivate the adoption of person-centred care in clinical practice. This could be expressed by decision-making at the macro (law, mission) level, which guides the meso (policies) and micro (routines) levels to adopt the scope and content of person-centred care in clinical practice. However, healthcare systems (Beveridge, Bismarck and out-of-pocket) have different structures and missions owing to ethical approaches. The quality of healthcare supported by evidence-based knowledge enables the establishment of a well-integrated phenomenon in European healthcare.

**Originality/value:**

Our findings clarify those countries using the Beveridge healthcare model rank higher on accepting/adopting the concept of person-centered care in discourse. To adopt the concept of person-centred care in discourse requires a systematic approach at all levels in the organisation—from the national (politicians) and regional (guideline) to the local (specific healthcare settings) levels of healthcare.

## 1. Background

Previous research has highlighted the importance of healthcare organisation, content of healthcare curriculum and financial systems used in healthcare ( Chenoweth *et al.* , 2019 ; Gyllensten *et al.* , 2019 ; Pirhonen *et al.* , 2017 , 2019 ; Rosengren *et al.* , 2018 ). The traditional model of healthcare is focused on diseases (medicine and natural science) and does not acknowledge patients' resources and abilities to be an expert in their own life based on their lived experiences ( CEN, 2020 ; IAPO, 2020 ). Risberg *et al.* (2006) emphasize the bio-medical framework as the dominating paradigm in medicine with epistemological roots in positivism. Such traditional biomedical tradition has been successful in producing useful medical knowledge, but they are not suited for ‘soft’ medicine like patient experiences or patient–doctor interaction, which are important elements of clinical medicine. According to Ekman *et al.* (2011 , 2015) , person-centred care recognises patients as persons with resources and abilities despite their disease. It includes listening to patient narratives, partnership and agreement between patients and healthcare professionals regarding healthcare activities based on patients' lived experiences, and evidence-based practice documented in a health plan ( GPCC, 2020 ). Moreover, research has shown that person-centred care is associated with shorter hospital stays, lower readmission rates, higher quality of care and satisfaction with healthcare ( Edvardsson *et al.* , 2011 , 2014 ; Ekman *et al.* , 2011 , 2015 ); Gyllensten *et al.* (2019) , McCance *et al.* (2013) , McCormack *et al.* (2010) , Pirhonen *et al.* (2019) . These advantages have led politicians and policymakers to have an increased interest in adopting and implementing person-centred care ( Swedish Agency for Health and Care Services Analysis, 2018a , b ). For example, Swedish law ( Swedish code of Statutes 2017:30, 2017 ) promotes inclusion of all citizens in health and well-being and recommends implementation of person-centred care. However, person-centred care is regarded as an unclear concept because of limited knowledge and understanding among healthcare professionals ( Lodge *et al.* , 2017 ; Moore *et al.* , 2017 ; Naldemirci *et al.* , 2017 ; Oppert *et al.* , 2018 ). Therefore, this study highlights areas for improvement, such as the education of healthcare professionals ( Rosengren *et al.* , 2018 ).

Britten *et al.* (2020) emphasised the use of practical insights from health professionals to facilitate successful clinical work for further adoption and implementation of person-centred care. They argue that research has only focused on the need to adopt and implement person-centred care, instead of actively involving all actors to develop a model of co-creation of care within the team (staff, patients, relatives and managers). Awareness about person-centred care and its effects on quality of health and well-being increase its implementation ( Alharbi *et al.* , 2014 ; Lydahl, 2017 ). According to Britten *et al.* (2020) , tools such as steering committees (ward managers, chief physicians) and change agents (assistant nurses, registered nurses, physicians) can act as facilitators to educate and implement person-centred care in day-to-day clinical practice. They stress that co-creation of care within the team (bedside) as well as within organisations is the core component to facilitate person-centred care—an ethical framework for improved health and well-being for all involved (patient, relatives, healthcare professionals and managers).

An integrative literature review including research from Australia, New Zealand, Canada, USA and Europe, described that no universally used definition of person-centred care exists. It highlighted three factors to understand and practice person-centred care: people, practice and power ( Byrne *et al.* , 2020 ). The review uncovered a malalignment between the concept of person-centred care and its operationalisation at the micro, meso and macro levels of the healthcare system. To our knowledge, the diffusion of person-centred care in Europe is still unknown, and it is unclear if the main construction of healthcare systems coincides with the occurrence of person-centred care. Therefore, this study aims to describe how person-centred care, as a concept, has been adopted into discourse in 23 European countries in relation to their healthcare systems (Beveridge, Bismarck, out of pocket).

## 2. Methods

### 2.1 Design

A systematic literature review ( Bettany-Saltikov and McSherry, 2016 ) inspired by the SPICE model ( Booth, 2004 ), including scientific (CINHAL, Medline, Scopus) and grey literature (Google), was used to describe the extent of person-centred care (scope and content) in Europe.

### 2.2 Data collection

To ensure variation of countries based on healthcare systems (Beveridge model, Bismarck model and out of pocket model) and localisation in Europe, the following countries were included: Belgium, Czech Republic, Denmark, Estonia, Finland, France, Germany, Greece, Hungary, Iceland, Ireland, Italy, Latvia, Malta, Norway, Poland, Portugal, Serbia, Slovenia, Spain, Sweden, the Netherlands and United Kingdom and the UK (England, Scotland, Wales, North Ireland). One inclusion criterion was the study of equal parts of the common healthcare systems. However, European countries practicing the main healthcare systems were unequal, resulting in inclusion of unequal number of healthcare systems—Beveridge model ( *n* = 12), Bismarck model ( *n* = 10) and out of pocket model ( *n* = 1). Former Eastern European countries were difficult to assort to the main healthcare systems. Another inclusion criterion was to include all parts of Europe, from the north, west and east to the Mediterranean area. Grey literature from Google and scientific literature published in peer-reviewed journals, citied in CINHAL, Medline or Scopus databases, between January 2010 and May 2020, from the above countries, containing the term “person-centred care” were included in the study. Exclusion criteria were papers written in a language other than English or Swedish and the use of words like person-centred care such as client-centred, medical-centred, family centred, patient-centred and people-centred care ( Table 1 ).

**Figure 1:**
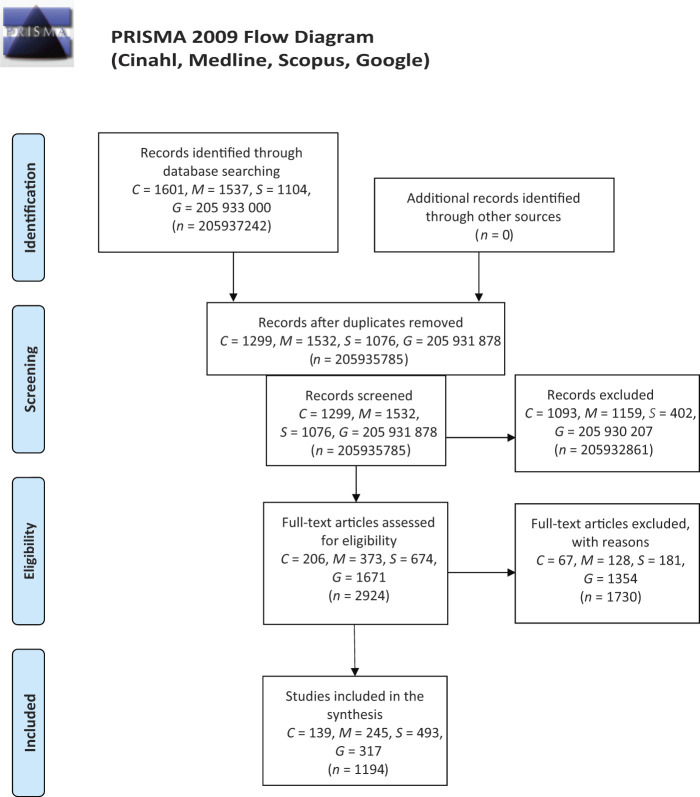
Search strategy and screening procedure according to SPICE

Literature was collected in four stages ( Figure 1a–1d ): identification of data (searching), screening of titles and abstracts to ensure that they met the inclusion criteria, discussion about eligibility and inclusion of data in the current study ( Bettany-Saltikov and McSherry, 2016 ). Suitable keywords (“person-centred”, “person-centred”, “person-centred”, “person-centred”, “person-centred”, “person centeredness”, “personcenteredness”, or “person centeredness”) for different databases (CINHAL, Medline, Scopus, Google) were identified. Data collection was conducted from November 2019 to May 2020 (CINHAL, *n* = 1,601; Medline, *n* = 1,537; Scopus, *n* = 1,104; Google, *n* = 205,933,000). Titles and abstracts were screened for aim and inclusion and exclusion criteria (CINHAL, *n* = 1,299; Medline, *n* = 1,532; Scopus, *n* = 1,076; Google, *n* = 205,931,878). Thereafter, selected documents (CINHAL, *n* = 206; Medline, *n* = 373; Scopus, *n* = 674; Google, *n* = 1,671) were screened and 1,194 documents were omitted (CINHAL, *n* = 139; Medline, *n* = 245; Scopus, *n* = 493; Google, *n* = 317).

### 2.3 Data analysis

The included literature (scientific/grey literature) from four databases (CINHAL, Medline, Scopus, Google) were analysed for content and scope of person-centred care in each country. Countries were grouped based on their specific healthcare systems (Beveridge, Bismarck, out of pocket) and geographic placement. Descriptive analysis ( Polit and Beck, 2017 ) was conducted for range (1–23), percentages (0%–100%) and valuation (5-point scale). Data from scientific (CINHAL, Medline, Scopus) and public literature (Google) were analysed using the following steps: (1) obtaining an overview of data regarding how person-centred care, as content, has been adopted in discourse; (2) data were analysed in parts (country and healthcare system), and the extent of person-centred care in relation to specific countries' healthcare systems were described within the included literature (CINHAL, Medline, Scopus and Google); (3) data were valued on a 5-point scale (1 = person-centred care is emphasised as something that needs to be established, 2 = thereafter person-centred care is going to be established in the country, 3 = person-centred care is an established phenomenon in some areas but not in others, 4 = person-centred care is established as a phenomenon, but improvements are needed within the country, 5 = person-centred care is well integrated in healthcare) in included documents (scientific, public) and (4) results are presented based on how the concept of person-centred care has been adopted into discourse based on different European countries' geographic placement and healthcare system (Beveridge, Bismarck, out of pocket).

### 2.4 Ethical considerations

The current systematic review is grounded in high ethical standards by following the ethical guidelines for human and social research ( Codex, 2020 ). All included data, especially data from scientific literature (CINHAL, Medline, Scopus), were assessed with respect to ethical considerations such as information, voluntariness and confidentiality to do no harm; to improve health and well-being ( Codex, 2020 ). Moreover, well-established scientific methods (systematic review and descriptive analysis) have been used to conduct scientifically sound studies ( Bettany-Saltikov and McSherry, 2016 ; Polit and Beck, 2017 ).

## 3. Findings

The results are presented by country, grouped by respective healthcare systems (Beveridge, Bismarck, out of pocket) and their geographic placement in Europe.

The most relevant content and scope of person-centred care was found in the UK ( *n* = 481, 40.3% of all literature), followed by Sweden ( *n* = 231, 19.3%). Thereafter, available literature dropped for the following 21 countries: the Netherlands ( *n* = 80, 6.7%), Ireland ( *n* = 79, 6.6%) and Norway ( *n* = 61, 5.1%). The remaining 18 countries (6–23) included 40 documents or less ( Table 2 ) within the scope of person-centred care. The bottom five countries include Poland (0.6%), Hungary (0.5%), Greece (0.4%), Latvia (0.4%) and Serbia (0%). Moreover, the most frequently used databases for person-centred care was Scopus (41.3%), followed by Google (26.5%), Medline (20.5%) and CINHAL (11.6%) ( Table 2 ).

Furthermore, in countries with the Beveridge healthcare system (12/23 = 0.5217, 52.2%), the term “person-centred care” was used in 85% of the papers (Google = 81%, CINHAL = 94%). Conflicting results were observed for countries with the Bismarck healthcare system (10/23 = 0.4347, 43.5%). The term “person-centred care” was used in 15% of the papers (CINHAL = 6%, Google = 18%). In countries with an out-of-pocket healthcare system, only Greece was included (4%) and resulted in 0.4% use of person-centred care (less scientific literature, Table 3 ).

Another way of presenting how common person-centred care is in the included countries, is by grouping them according to their geographic location (east, north, south and west). Person-centred care is the most common (50.6%) in the west of UK (England, Northern Ireland, Scotland, Wales) and Ireland; second (27.5%) in the north of Scandinavian countries (Sweden, Norway, Denmark, Finland, Iceland), followed by central Europe (11%; the Netherlands, Germany, Belgium), south (8%) of Europe (Spain, Italy, Malta, Portugal, France, Slovenia, Greece and Serbia) and finally in the east of Europe (3%; Estonia, Czech Republic, Poland, Hungary, Latvia).

Moreover, the included countries ( *n* = 23) varied in the number of documents, content and scope of person-centred care, which were valued on a 5-point scale as follows: (1) person-centred care is emphasised as something that needs to be established; (2) person-centred care is going to be established in the country; (3) person-centred care is an established phenomenon in some areas but not in others; (4) person-centred care is established as a phenomenon and there are improvements within the country; and finally, (5) person-centred care is a well-established phenomenon and well-integrated part of healthcare. Countries with the Beveridge system (12/23 = 0.52, 52%) used more person-centred care (7/10 countries) compared with countries with the Bismarck healthcare system ( Table 4 ).

The highest number of documents available on the content and scope of person-centred care was found for the UK (England, Scotland, Wales and Northern Ireland), with a value of 4.35 (1–5), for the establishment of person-centred care, followed by Sweden (4.42; Table 4 ). The Netherlands (2.95) was placed third with two other countries within the Bismarck system (3/10 countries, 30%). Germany (2.38) placed sixth, and Belgium (2.53) tenth. Lesser documents (PCC) were found in other countries, seven out of ten countries within the Bismarck system (Estonia at place 14; Slovenia, 16; Czech Republic, 17; France, 18; Poland, 19; Hungary, 20 and Serbia, 23), two countries with the Beveridge system (Iceland at place 15, Latvia at 22) and Greece (place 21) with the out-of-pocket healthcare system ( Table 4 ).

## 4. Discussion

**Figure 2:**
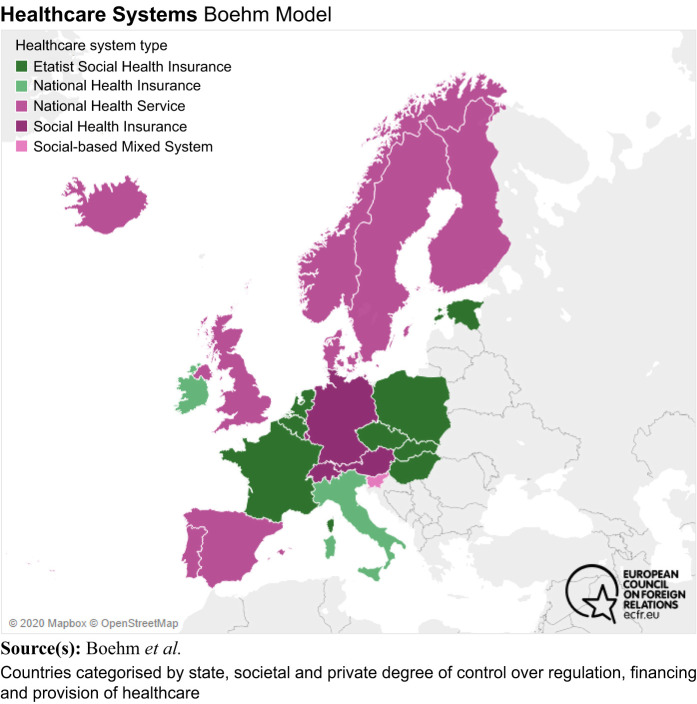
Overview of European countries due to provision of healthcare

The current study describes the extent of person-centred care in 23 European countries in relation to their healthcare systems (Beveridge, Bismarck and out of pocket). The main difference in using the concept of person-centred care in research and grey literature was that between Beveridge and Bismarck healthcare models. In literature and documents, this concept appeared more frequently in countries using the Beveridge model than the Bismarck model. Or *et al.* (2010) suggest that the main differences in underlying values are universality and equity in countries with the Beveridge model versus plurality, liberty and solidarity in countries with the Bismarck model. Even if the Beveridge type of healthcare is available for all inhabitants, the possibility of making choices is limited, while the Bismarck model offers a variety of providers, but the distribution of healthcare is not always equal ( Torbica *et al.* , 2018 ). This can be illustrated by the Boehm models ( European Council on Foreign Relations, 2020 ), where countries are categorised by state societal and private degrees of control over regulation, financing and provision of healthcare. In Figure 2 , green and dark pink illustrate insurance systems (mainly Bismarck), while medium pink illustrates the types of national health services (mainly Beveridge).

The results show that person-centred care was most adopted into discourse in the UK (England, Scotland, Wales and Northern Ireland), followed by Sweden, the Netherlands, Ireland and Norway, compared with Poland, Hungary, Greece, Latvia and Serbia. Moreover, seven out of ten countries with the Beveridge model used the concept of person-centred care in scientific literature, as opposed to the Bismarck model, where the concept was mostly supported by grey literature. Since only Greece (placed 21) practices an out-of-pocket healthcare system, it is difficult to draw conclusions regarding the scope and content of person-centred care in relation to this healthcare system.

The Beveridge healthcare system shows the majority using the concept of person-centred care (84% vs. 16%) compared with the Bismarck healthcare system, which has a variation of scope and content of person-centred care, based on geographic locations (east, north, south and west) of the European countries included in this study. Since person-centred care is seen in 40.3% of the documents available for Western European countries (the UK and Ireland), it can be considered an established and well-integrated phenomenon, especially in areas of elderly care ( McCormack *et al.* , 2010 ). Similar results were observed for some of the Scandinavian countries, such as Sweden (19.3%). However, this result is lower for Northern Europe, Norway (5.1%), Denmark (2.6%), Finland (1.5%) and Iceland (0.8%), and Central Europe, the Netherlands, Germany and Belgium (6.6 %–6.6%). The following discussion is focused on why there is a variation in adopting the concept of person-centred care: the relationship between person-centred care and healthcare system or vice-versa. One reason could be that in the UK and Sweden, the government decided to implement person-centred care based on evidence-based research regarding the efficiency of this healthcare practice ( Edvardsson *et al.* , 2011 , 2014 ; Ekman *et al.* , 2011 , 2015 ; Gyllensten *et al.* , 2019 ; McCance *et al.* , 2013 ; McCormack *et al.* , 2010 ; Pirhonen *et al.* , 2019 ). Therefore, regulations and laws (e.g. the Swedish Code of Statutes, 2017:30 ) focus on shared decision-making in line with person-centred care ( GPCC, 2020 ; Ricoeur, 1992 ). Moreover, patient unions ( IAPO, 2020 ; OECD, 2017 ) influence healthcare in favour of acknowledging patients as experts in their own lives through their lived experiences and partners in their own healthcare. This approach is grounded in democracy, covered in international human rights law, and is based on active involvement in health and well-being ( UN, 2020 ).

However, in the south (except Spain) and east of Europe, person-centred care seems to be less common based on the content available. The reasons for this outcome need to be examined in other studies. It may be due to cultural differences in the society and healthcare sectors. Another reason can be difficulties experienced in the rapidly changing processes of the healthcare sector from a Semashko, and post-Semashko-type of healthcare systems in former Eastern Europe ( Kaminska *et al.* , 2021 ).

The current results could also be discussed in relation to public versus private/insurance-based healthcare due to high quality of healthcare, democracy, human rights, as well as committed patient associations and unions, which are in line with the ethical approach of person-centred care ( IAPO, 2020 ; OECD, 2017 ). Public funded healthcare and taxations are used in the UK, Scandinavian countries and Spain—countries ranked higher for adopting the discourse of person-centred care. This could be because healthcare is organised by the government and selected politicians, who make decisions on the framework for specific healthcare settings, such as laws, budgets and goals. Therefore, it is easier to call upon changes because evidence-based knowledge is used as the foundation for developing laws and policies. Moreover, basic democratic assumptions are used at different levels of community/state ( OECD, 2017 ; Swedish Agency for Health and Care Services Analysis, 2018a , b ). Participation and shared decision-making are used to influence the quality of care. Person-centred care is one way to improve healthcare, which is in line with research and goals based on patient associations ( Alharbi *et al.* , 2014 ; Britten *et al.* , 2020 ; IAPO, 2020 ).

The traditional healthcare model focuses more on patients' disease and reasons behind the occurrence of suffering instead of patients' resources/abilities and health and well-being ( Alharbi *et al.* , 2014 ). The traditional biomedical approach, assume a disease as deviations from quantifiable biological variables of normality. Such an approach is only concerned with organic malfunctions and measurable aspects of illness. However, healthcare professionals need to acknowledge patients as human beings with experiences, desires and wishes, who can take responsibility for their health and well-being. However, healthcare professionals are resistant to these changes. Evidence-based knowledge is an important motivator for improvement in clinical practice ( Alharbi *et al.* , 2014 ; Rosengren, 2016 ; Wranik, 2012 ). A systematic approach ( Britten *et al.* , 2020 ) regarding the scope and content of person-centred care (organisational and professional) is one tool that facilitates successful clinical work. Research has focused on the need of healthcare systems to adopt and implement person-centred care; however, research should also focus on co-creation of healthcare (staff, patients, relatives and managers). Evidence-based knowledge regarding high-quality care is important for facilitating changes in the healthcare system. Research ( Ekman *et al.* , 2011 , 2015 ; GPCC, 2020 ) show that listening carefully to patients' narratives and agreement in partnership (between the patient and healthcare professionals) regarding essential matters documented in a health plan, facilitates efficient healthcare ( Gyllensten *et al.* , 2019 ; Pirhonen *et al.* , 2019 ). A key factor that facilitates knowledge translation and the use of limited resources is co-creation of care as synergy leads to shorter hospital stays, lower readmission rates, higher quality of care and patient satisfaction with healthcare ( Edvardsson *et al.* , 2011 , 2014 ; Ekman *et al.* , 2011 , 2015 ; Gyllensten *et al.* , 2019 ; McCance *et al.* , 2013 ; McCormack *et al.* , 2010 ; Pirhonen *et al.* , 2019 ).

It may be more difficult to adopt and implement innovations such as person-centred care in private/insurance-based healthcare systems because of the autonomous nature of care providers without a common organisational umbrella. For example, Germany has over 200 insurance companies covering healthcare. This could lead to difficulties regarding the national implementation of a specific innovation or care model ( Härter *et al.* , 2017 ). Another reason is that there are independent caregivers in different departments/settings in the public sector. Adoption of person-centred care is possible with systematic frameworks supported by regulations and laws that Britten *et al.* (2020) highlight, together with co-creation of care between patients, healthcare professionals, managers and politicians. This systematic point of view is supported by strong patient unions as well as evidence-based knowledge developed by research regarding the scope and content of person-centred care, which can be a motivator for the national adoption and implementation of person-centred care.

According to the results, neighbouring countries seem to influence each other ( Alharbi *et al.* , 2014 )—UK–Ireland, and Scandinavian countries organise healthcare in the same way. For example, the Nordic model is funded by taxes with similarities regarding democratic aspects and the healthcare system—an umbrella that facilitates person-centred care. However, results indicate that person-centred care is an unclear phenomenon among health professionals. Therefore, future research regarding person-centred care needs to focus on clinical implications, as well as a systematic point of view when person-centred care is adopted and implemented in healthcare systems across Europe ( Britten *et al.* , 2020 ). The future goal is to increase awareness about how person-centred care can be used in clinical practice to improve health and well-being ( Ekman *et al.* , 2011 ; Røen *et al.* , 2018 ; Vassbø *et al.* , 2019 ).

## 5. Limitations

There are limitations to this systematic literature review. First, the term “person-centred care” was used to map person-centred care in different European countries, where the scope and content of person-centred care could be named differently, as mentioned in the exclusion criteria. Therefore, documents could have been lost. Future research should include a wider range of terms. Second, only 23 out of the 49 European countries were included, investigating only a part of the European perspective. Another limitation is that only one country used an out-of-pocket healthcare system; therefore, the overall conclusions regarding this healthcare system are limited. The current study indicates that healthcare systems and geographic placement influence the scientific soundness of research and quality of care ( Bettany-Saltikov and McSherry, 2016 ; Polit and Beck, 2017 ). However, the current study should consider the above limitations. Future studies are required to confirm these results.

## 6. Conclusion

Our findings clarify those countries using the Beveridge healthcare model rank higher on accepting/adopting the concept of person-centered care in discourse. To adopt the concept of person-centred care in discourse requires a systematic approach at all levels in the organisation—from the national (politicians) and regional (guideline) to the local (specific healthcare settings) levels of healthcare. Evidence-based knowledge as well as national regulations regarding person-centred care are important tools to motivate the adoption of person-centred care in clinical practice. This could be expressed by decision-making at the macro (law, mission) level, which guides the meso (policies) and micro (routines) levels to adopt the scope and content of person-centred care in clinical practice. However, healthcare systems (Beveridge, Bismarck and out-of-pocket) have different structures and missions owing to ethical approaches. The quality of healthcare supported by evidence-based knowledge enables the establishment of a well-integrated phenomenon in European healthcare.

## Figures and Tables

**Table 1:** Search terms for different databases

Search terms	Medline	Cinahl	SCOPUS	Google
Health system	(1) Beveridge ( *n* = 12)	(1) Beveridge ( *n* = 12)	(1) Beveridge ( *n* = 12)	(1) Beveridge ( *n* = 12)
	(2) Bismarck ( *n* = 10)	(2) Bismarck ( *n* = 10)	(2) Bismarck ( *n* = 10)	(2) Bismarck ( *n* = 10)
	(3) OOP ( *n* = 1)	(3) OOP ( *n* = 1)	(3) OOP ( *n* = 1)	(3) OOP ( *n* = 1)
“Person centered” OR “person centred” OR “person-centered” OR “person-centred” OR “person centeredness” OR personcenteredness OR “person centredness”	(1) 1,317	(1) 1,298	(1) 881	(1) 125,384,000
	(2) 213	(2) 298	(2) 213	(2) 65,349,000
	(3) 7	(3) 5	(3) 10	(3) 15,200,000
Total numbers	1,537	1,601	1,104	205,933,000

**Table 2:** Overview of content and scope of person-centred care due to countries and databases

Countries	Scopus	Google	Medline	Cinahl	Total
	*n* = 493 41.3%	*n* = 317 26.5%	*n* = 245 20.5%	*n* = 139 11.6%	*N* = 1,194 100%
1. UK (England, Scotland, Wales, North Ireland)	168	148	90	75	481/1,194 40.3%
2. Sweden	119	12	68	32	231/1,194 19.3%
3. The Netherlands	40	16	18	6	80/1,194 6.7%
4. Ireland	33	22	15	9	79/1,194 6.6%
5. Norway	36	9	14	2	61/1,194 5.1%
6. Germany	22	5	13	0	40/1,194 3.4%
7. Spain	15	6	9	5	35/1,194 2.9%
8. Denmark	11	9	7	4	31/1,194 2.6%
9. Italy	11	9	0	0	20/1,194 1.7%
10. Belgium	9	3	5	2	19/1,194 1.6%
11. Finland	9	4	1	4	18/1,194 1.5%
12. Portugal	2	13	0	0	15/1,194 1.3%
13. Malta	0	14	0	0	14/1,194 1.2%
14. Estonia	2	10	0	0	12/1,194 1%
15. Iceland	3	5	1	0	9/1,194 0.8%
16. Slovenia	3	5	1	0	9/1,194 0.8%
17. Czech Republic	1	7	1	0	9/1,194 0.8%
18. France	4	3	1	0	8/1,194 0.7%
19. Poland	2	4	1	0	7/1,194 0.6%
20. Hungary	1	5	0	0	6/1,194 0.5%
21. Greece	2	3	0	0	5/1,194 0.4%
22. Latvia	0	5	0	0	5/1,194 0.4%
23. Serbia	0	0	0	0	0/1,194

**Table 3:** Overview of valued of content and scope of person-centred care due to health system

Countries	Total documents Cinahl, Google, Medline, Scopus	Value (1–5) *C* =Cinahl *G* = Google *M* = Medline *S* =Scopus *T* = Total	Health system (1) Beveridge 12/23 = 52.2% (2) Bismarck 10/23 = 43.5% (3) OOP 1/23 = 4%
1. UK (England, Scotland, Wales, North Ireland)	481/1,194 40.3%	*C* = 3.96	1
		*G* = 4.33	
		*M* = 4.24	
		*S* = 3.74	
		*T* = 4.07	
2. Sweden	231/1,194 19.3%	*C* = 3.53	1
		*G* = 4.41	
		*M* = 3.18	
		*S* = 3.43	
		*T* = 3.38	
3. The Netherlands	80/1,194 6.7%	*C* = 3.33	2
		*G* = 4.25	
		*M* = 2.72	
		*S* = 2.8	
		*T* = 3.27	
4. Ireland	79/1,194 6.6%	*C* = 3.57	1
		*G* = 4.04	
		*M* = 3.8	
		*S* = 2.84	
		*T* = 3.56	
5. Norway	61/1,194 5.1%	*C* = 4	1
		*G* = 3.5	
		*M* = 2.5	
		*S* = 3.25	
		*T* = 3.31	
6. Germany	40/1,194 3.4%	*C* = 1	2
		*G* = 4.6	
		*M* = 2	
		*S* = 2.77	
		*T* = 2.59	
7. Spain	35/1,194 2.9%	*C* = 2	1
		*G* = 4	
		*M* = 3.11	
		*S* = 2.2	
		*T* = 2.82	
8. Denmark	31/1,194 2.6%	*C* = 3	1
		*G* = 4.4	
		*M* = 3.14	
		*S* = 2.91	
		*T* = 3.36	
9. Italy	20/1,194 1.7%	*C* = 3.33	1
		*G* = 3.11	
		*M* = 1	
		*S* = 2.71	
		*T* = 2.53	
10. Belgium	19/1,194 1.6%	*C* = 2.5	2
		*G* = 5	
		*M* = 3	
		*S* = 2.11	
		*T* = 3.15	
11. Finland	18/1,194 1.5%	*C* = 3,5	1
		*G* = 4	
		*M* = 5	
		*S* = 3.11	
		*T* = 3.9	
12. Portugal	15/1,194 1.3%	*C* = 1	1
		*G* = 3.92	
		*M* = 1	
		*S* = 3	
		*T* = 2.23	
13. Malta	14/1,194 1.2%	*C* = 3.33	1
		*G* = 4.28	
		*M* = 1	
		*S* = 1	
		*T* = 2.4	
14. Estonia	12/1,194 1%	*C* = 1	2
		*G* = 3.7	
		*M* = 0	
		*S* = 1.5	
		*T* = 1.55	
15. Iceland	9/1,194 0.8%	*C* = 1	1
		*G* = 4.5	
		*M* = 4	
		*S* = 2.33	
		*T* = 2.95	
16. Slovenia	9/1,194 0.8%	*C* = 3.33	2
		*G* = 3.6	
		*M* = 2	
		*S* = 2.66	
		*T* = 2.89	
17. Czech Republic	9/1,194 0.8%	*C* = 1	2
		*G* = 3.42	
		*M* = 2	
		*S* = 2	
		*T* = 2.10	
18. France	8/1,194 0.7%	*C* = 1	2
		*G* = 4	
		*M* = 3.5	
		*S* = 3.25	
		*T* = 2.94	
19. Poland	7/1,194 0.6%	*C* = 3.33	2
		*G* = 3	
		*M* = 2	
		*S* = 3.5	
		*T* = 2.96	
20. Hungary	6/1,194 0.5%	*C* = 0	2
		*G* = 4.25	
		*M* = 1	
		*S* = 3	
		*T* = 2.06	
21. Greece	5/1,194 0.4%	*C* = 0	3
		*G* = 5	
		*M* = 1	
		*S* = 3	
		*T* = 2.25	
22. Latvia	5/1,194 0.4%	*C* = 0	1
		*G* = 2.2	
		*M* = 0	
		*S* = 0	
		*T* = 0.55	
23. Serbia	0/1,194	0	2
*Total*	*1,194*	*2.64*	*(1) 2.92 (0.55–4.07) n = 999/12*
			*(2) 2.35 (0–3.27) n = 190/10*
			*(3) 2.25 n = 5/1*

**Table 4:** Overview of person-centred care due to health system and databases

Healthcare system	Google	Scopus	Medline	Cinahl	Total
*Beveridge;* 12/23 = 0.5217 52.2%	256	407	205	131	999
Denmark	256/317 = 0.81 81%	407/493 = 0.83 83%	205/245 = 0.84 84%	131/139 = 0.94 94%	999/1,194 = 0.84 84%
Finland Ireland Iceland					
Italy					
Latvia Norway Malta Portugal Spain, Sweden					
UK i.e. Scotland Wales Northern Ireland England					
*Bismarck* 10/23 = 0.4347 43.5%	58	84	40	8	190
Belgium Czech Republic Estonia France Germany	58/317 = 0.18 18%	84/493 = 0.17 17%	40/245 = 0.16 16%	8/139 = 0.06 6%	190/1,194 = 0.16 16%
Hungary Netherlands Poland Serbia Slovenia					
*OOP* 1/23 = 0.04% 4%	3	2	0	0	5
Greece	3/317 = 0.01 1%	2/493 = 0.004 0.4%	0%	0%	5/1,194 = 0.004 0.4%
